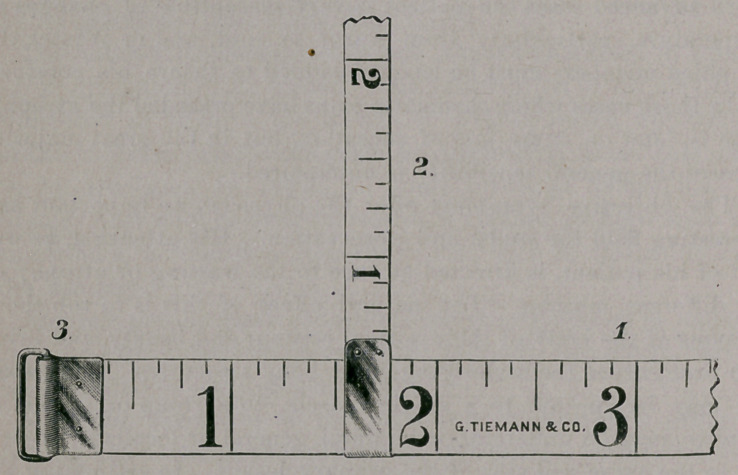# Muscular Atrophies: A Clinico-Pathological Study1Read before the Buffalo Medical Club, Nov. 5, 1890. Published by request.

**Published:** 1891-04

**Authors:** William C. Krauss

**Affiliations:** Lecturer on Pathology in the Niagara University Medical College, Buffalo, N. Y.


Buffalo Medical and Surgical Journal

VOL. XXX. APRIL, 1891. NO. 9.

### Original Communications.

### MUSCULAR ATROPHIES: A CLINICO-PATHOLOGICAL STUDY.[1]

BY WILLIAM C. KRAUSS, M. D.,

Lecturer on Pathology in the Niagara University Medical College, Buffalo, N. Y.

ATROPHY is a retrogressive change taking place in parts originally well formed and well grown. Muscular atrophy is a retrogressive change taking place in the muscular fiber, causing a diminution in its bulk and substance, thereby rendering it incapable of performing its normal physiological function. Muscular atrophy must not be confounded with hypoplasias or aplasias, but only when occurring in muscles once able to perform work measured by their development and vitality. On the other hand, we must keep distinct and separate a retrogressive movement occurring when the active cell growth is no longer predominant, but has been succeeded by a period of involution or cell decay; this we designate as senile wasting, or physiological retrogression or active atrophy.

Another class of atrophies, which are more physiological than pathological, are the passive atrophies. These are occasioned by diminished nutrition, general anemia, defective assimilation, febrile processes, starvation, etc. Local atrophies, due to mechanical hindrances, injury to the tissue or through some interference with the circulation, are also examples of passive atrophies.

Analogous retrogressive processes may occur prematurely under pathological conditions, and to these I wish to call your attention this evening.

In 1848, Duchenne (*de Boulogne, France,*) presented a memoir to the Institute on the Symptomatology, Differential-diagnosis and Pathogenesis of progressive muscular atrophy. Aran, a co-worker

1. Read before the Buffalo Medical Club, Nov. 5, 1890. Published by request.

of his, at the Hôpital de la Charité, Paris, appropriated to himself, in great measure, the deductions and facts collected by Duchenne, and published the first paper on this subject in 1850. Prior to this ime, Sir Charles Bell made some observations on chronic muscular atrophy and was able to differentiate it from primary paralysis. The observations of Duchenne and Aran gave a new impetus to the study of this affection, and since that time important discoveries and researches have been instituted, among others, by Cruveilhier Charcot, Luys, Landouzy, Dejerine and the school of the Salpêtrière in France; Griesenger, Cohnheim, Leyden, Liebreich and Erb in Germany; Lockhart–Clarke, Gowers and Tooth in England; Hammond, Sachs and others in America.

At the present time, although the study of this affection is well formulated, and the views held by different observers have become more or less harmonized, yet the resemblances between the different forms cause some difficulty in rendering a proper diagnosis and a consequent prognosis.

*Histology.*— A striated muscle is composed of a number of bundles, surrounded by a layer of areolar tissue, the external perimysium. Eachbundle or fasciculus, enveloped by a thin delicate membrane, the internal perimysium, is composed of bundles of fibers, separated from each other by a delicate connective tissue, the endomysium. These fibers are arranged parallel to each other, are from an inch to one and a half inches in length, and are united either to the tendons or aponeuroses, or else connected with the adjacent fiber.

Each fiber is composed of a number of filaments or fibrillæ, inclosed in a transparent homogeneous membrane, designated by Bowman the sarcolemma. In the mammalia, elongated nuclei are present on the internal surface of this membrane. The primitive fibers are cylindrical or prismatic in form, about one-four-hundredth of an inch in breadth, and their length depends not so much the length of the muscle, as upon the arrangement of the tendons. They are marked by transverse and longitudinal lines or striæ, giving them a characteristic, striated or striped appearance. I will not take up the histology of the primitive fibrillæ, but will limit myself to the primitive fiber. (See Plate, Fig. I.)

Each fiber has a vascular and nervous supply, the former being furnished by the ramifications of the capillaries, running parallel between the fibers. The nervous supply is from the motor nerves, and their termination in the muscle has been the subject of much controversy. The motorial end plates of Kühne or nerve hillocks of Doyère are generally recognized by most recent observers. The nerve terminates below the sarcolemma, where the medullary sheath becomes blended with it, forming a plate or plaque which is raised somewhat from the fibers, but never encircles it. The axis cylinder is distributed to this plaque, but does not penetrate the interior of the fiber. The origin of the efferent or sensory nerve fibers in the muscle is still a matter of uncertainty.

Degenerative changes must, therefore, be produced by a lesion to the efferent nerves or nerve centers higher up, by some nutritive disturbance, or else through primary disintegration of the protoplasm of the muscle itself.

The two pathological conditions of the muscle which I wish to consider are, simple degenerative atrophy and fatty infiltration, —Lipomatosis.

*Pathology.*—The most common form is the simple degenerative atrophy, as typified in progressive muscular atrophy. To the naked eye there is little to be seen save the diminution in size, and the pale, pinkish hue of the fibers; to the touch, a soft, spongy feel, with occasional cord-like prominences, instead of a firm resistant mass. The entire muscle, if carefully removed, will be found shorter than normal owing to the contraction of the interstitial connective tissue. Under the microscope the condition is as follows: If the atrophy is not too far advanced, the fibers retain their normal appearance—transverse and longitudinal striation,—but are somewhat narrower. As the process advances, the fibers split up into longitudinal fibrillæ, or transversely into discoid masses and then gradually disappear. In other cases fatty and vitreous degeneration may occur, and the fiber then has the appearance of a sheath containing a clear material with some fat globules. The intensity of this process is not the same throughout the muscle, patches of healthy fibers may be found surrounded by others in different stages of atrophy. Proliferative changes occur in the nuclei of the muscular fibers, and may lead to a new cell growth within the sarcolemma, replacing the contractile substance. Proliferation of the interstitial tissue also occurs and to such an extent as to separate the neighboring fibers. The entire muscle may, in fact, be converted into bands of connective tissue with some fat globules interposed between the separate layers. (See Plate, Fig. II.)

Fatty infiltration and degeneration of the muscular fiber is met with less frequently. Here hyperplasia of the interstitial connective tissue and fatty infiltration follow closely upon the wasting of the muscle, and cause either no apparent change or else a slight increase in its volume. The muscle appears pale, yellowish, has a greasy feel, and resembles closely, not only macroscopically but also microscopically, a lipoma, or, better, a myo-lipoma. Under the microscope, the large, round, yellowish cells, with dark borders, make up the greater portion of the tissue. Here and there a muscular fiber, with its transverse and longitudinal striation still intact, may be observed. (See Plate, Fig. III.)

The interstitial connective tissue is much increased in volume with proliferation of its nuclei. The substitution of fat may be so pronounced as to give the muscle an hypertrophied appearance, and hence the denomination pseudo-hypertrophy, given this affection by Duchenne in 1861. In some forms of dystrophia, the muscular fiber may be even increased in volume, giving rise to real hypertrophy, a condition sometimes met with in idiopathic muscular atrophy.

Another pathological condition, sometimes found in muscles, especially after infective processes as typhoid, diphtheria, variola, etc., is hyaline or waxy degeneration.

With the exception of the primitive or idiopathic group of muscular atrophies, or those in which the lesion is probably due to some metamorphosis in the protoplasm itself, the pathology is no longer debatable. In this group the muscular changes are not dependent upon any neuropathic lesion, as nerve, cord and brain, after repeated examinations have been found intact.

*The Symptoms* may be classified as subjective and objective Subjective: The advent of muscular atrophy in many cases, and especially in those of so-called rheumatic origin, is ushered in by some localized, deep-seated, aching pain, to which little attention is paid. In others, some slight sensory disturbance, as a feeling of numbness, heaviness or sharp lancinating pains as in neuritis may precede the atrophy, while in many no warning whatever is given of the enfeeblement which is soon to occur. Generally the first thing that attracts the patient's attention is the inability to execute certain movements, which, but a short time ago, he was able to carry out with ease and dexterity. If he be an artisan and the atrophy begins in the muscles of the hand, as it so often does, the weakness will soon incapacitate him for his work; if a laborer and the atrophy first affects the shoulder muscles, or muscles of the back, or if a pedestrian and the peroneal muscles succumb early, he is soon made cognizant of some loss of power, which to him remains for some time unaccountable. Sometimes this weakness is ascribed to overwork, exhaustion, or fatigue, and the usual remedy — rest — fails to restore to the former condition. I have met patients in clinics, especially females, in whom atrophy of the muscles of the hand and arm had existed for years, and attention first called to it by the physician while examining for some other ailment.

In advanced cases the patient is very susceptible to changes of temperature, particularly from warm to cold, and in Winter the atrophied members must be heavily padded to insure his comfort.

In those cases where rheumatic pains have preceded the atrophy, there is, later on, some loss of sensation, but in the great majority of cases the general sensibility is unimpaired.

The objective symptoms offer the physician an important and interesting field for study and observation. His attention, as was that of his patient, is directed at once to the wasting or atrophy of the different muscles. The natural effect of this is to rob those portions of the body of their normal contour and beauty, and bring into prominence the underlying hard bony structures. This wasting may be limited to a single muscle, to a group or system of muscles, may be unilateral or bilateral, general or localized, according to the cause and seat of the primary lesion. In estimating the extent of the atrophy, some more definite means are necessary than merely the sight or touch — and the tape measure is called into service.

A tape measure (p. 518) which seems to answer every purpose and which has been cordially received by many neurologists, was described by me in the April (1890) number of this journal.[1]

It consists of a tape (1) thirty-six inches long and one-half inch wide. The English scale is graduated on one side and the metric scale on the other. The head is supplied with a swivel (3), through which passes the free end of the tape, permitting of uniform tension, greater accuracy in reading, and of its being held with one hand.

The second tape (2) is eighteen inches long and one-quarter inch wide, and is provided with a sliding head, through which the first tape passes. This tape is, therefore, at right-angles to, and movable upon, the first tape. It is also graduated after the English and metric scales. The object of this tape is to ascertain at what distance from a certain fixed bony point the first tape has been

1. See also the *Journal of Nervous and Mental Diseases*, 1890; p. 128.

applied, so that on succeeding occasions the measurement may be taken at the same point. To illustrate: If the tape (1) be applied to the arm at a distance of five inches from the internal condyle of the humerus (reckoned by means of tape 2), it is obvious that on succeeding occasions, or in comparison of the two extremities, the tape (1) must be applied at exactly the same point, thus excluding all possible chance of error.

The atrophy of muscular fibers and the hyperplasia of the connective tissue lead to contraction of the latter, and permanent contractions and distortions of the body and extremities result; the same is produced if a system of muscles is affected and the opponents, remaining intact, predominate. The peculiar shape of the hand in the Duchenne–Aran type, sometimes called *main de griffe*, the turkey gait in the myopathic forms, etc., are examples of this kind.

The integument of the atrophied members has a shrivelled purplish appearance, and the finger nails lose their pinkish tint. Other trophic disturbances, except in atrophies due to a neuritis, are wanting.

In many cases a fibrillary contraction, wave-like in appearance, propagated in the direction of the fibers may be observed occurring either spontaneously or by gently tapping the muscle. This fibrillation, as it is termed, is of short duration, returns after an interval of a few seconds, may be limited to a muscle, or part of a muscle, or may extend over the whole of the affected part or member. It is not pathognomonic of progressive muscular atrophy, as was formerly supposed, but has been observed in other affections of the muscular system, and even in the healthy muscle.

Loss of Myotatic Irritability: Tapping a healthy muscle produces a slight contraction of the fibers, which calls forth the performance of its function. In the diseased muscle the reflex arc is broken, the centripetal-sensory path remaining undisturbed, while the centrifugal-motor path is broken. The loss of tendon reflexes, in some forms, occurs quite early, even before any serious damage has taken place in the muscular fibers. The patellar and elbow reflexes are the ones most generally examined.

Electrical Irritability: To Duchenne (*de Boulogne*) must be given the credit of having first employed electricity as a diagnostic and therapeutic agent. His method of localizing the electrical current, published in 1850, has served as the foundation for all later electrical researches in medicine. The elder Remak appeared against him, disputing some of his conclusions, particularly as to whether the contraction of the muscle was produced by irritating the bulk of the muscle, or the entrance of the motor nerve into the muscle. Von Ziemssen, taking advantage of this breach, made experiments upon dying patients, and, by careful dissection afterward, discovered that the motor points were those points where the motor nerve approached nearest the surface. 1857. The natural law of muscular contraction under the influence of the galvanic or faradic current, shows the superiority of the cathode pole over the anode, the contractions being short, sharp, and quick. The wasted muscle presents changes of electrical irritability dependent upon the degree and extent of the degeneration. Erb and V. Ziemssen conducted a series of experiments upon diseased muscles, and arrived at practically the same conclusions at exactly the same time—1868.

Their law, called the Entartungs Reaction, reaction of degeneration, is as follows: First degree, or partial reaction; faradic and galvanic nerve irritability preserved, but weakened; faradic and galvanic muscle irritability preserved, but the contractions, instead of being short, sharp and quick, are slow and vermiform. In the second degree, or complete degenerative reaction, the galvanic and faradic nerve irritability and faradic muscle irritability are lost, but the galvanic muscle irritability is increased. The action of the poles is, however, reversed, the anode closure contraction being greater than the cathode closure, and thirdly, the contractions are slow and vermiform. In the third degree, or severe type, there is entire loss of galvanic and faradic nerve and muscle irritability.

In progressive muscular atrophies, any one of these three degrees may be present, according to the seat and character of the primary lesion. Of these symptoms, the wasting and weakness are the only ones which are truly pathognomonic. The others, which are characteristic, are present in some forms of muscular atrophy, and absent in others.

There still exists some difference of opinion regarding the different forms, and each writer has a classification patterned after his own ideas. In fact, neurology is abreast with the other specialties. No one can claim to be an ophthalmologist without having invented an ophthalmoscope; no one can lay claim to be an obstetrician without having invented an obstetrical forceps; and in neurology, a classification of the muscular atrophies is the criterion. Far from drawing any inferences, nevertheless I have made a classification, which is nothing more than a compilation after the different authorities, based upon anatomo-pathological investigations.

CLASSIFICATION.

- MUSCULAR ATROPHY.

  - Physiological.

    - Active.

      - Senile Wasting.

    - Passive.

      - Diminished Nutrition.

      - Defective Assimilation.

      - Febrile Processes.

      - Direct Traumatism, etc.

  - Pathological.

    - Functio-Lesio.

      - Anchyloses.

      - Surgical Appliances.

      - Hysterical Contractures, etc.

    - Neuropathic.

      - Spontanic, Secondary, Traumatic, etc.

      - Toxic.

      - Infective Processes.

      - Arthritic.

    - Myopathic.

      - Scapulo-Humeral. (Erb's Juvenile Form.)

        - *a.* Facio-Scapulo-Humeral. (Landouzy-Dejerine.)

        - *b.* Peroneal. (Leyden's Hereditary Form.)

      - Paralysis Pseudo-Hypertrophic.

    - Myelopathic.

      - Acute.

        - Poliomyelitis acuta Infantilis.

        - Poliomyelitis acuta Adultorum.

      - Chronic.

        - Protopathic.

          - Hand Type. (Duchenne-Aran.)

          - Peroneal Type. (Charcot-Tooth.)

        - Deuteropathic.

          - Amyotrophic Lateral Sclerosis.

          - Syringomyelia.

          - Gliomatous Growths.

          - Locomotor Ataxia.

          - Multiple Sclerosis.

          - Diffuse Myelitis.

          - Myelo-Myelitis, etc.

    - Cerebropathic.

      - Cerebral Palsies.

        - Hemiplegia.

        - Diplegia.

        - Paraplegia.

Pathological atrophies are either atrophy of inaction (functio-lesio), or tropho-neurotic.

Functional activity and nutrition progress hand in hand, and, vice versa, decline. Hence, when functional activity in the cells is diminished or absent, that organ begins to waste or atrophy. Under this head we meet atrophies due to anchyloses, surgical appliances, hysterical contractions, etc.

There is no specific lesion, the atrophy being produced by the inaction of the muscles through some impediment to their normal activity. We have all met examples of this kind ; the atrophy is always local, non-inflammatory, and on resumption of motion the atrophy disappears.

The largest and most important class of atrophies is the trophoneurotic, or those dependent upon some lesion of either nerve, muscle, cord, or brain, and designated as neuropathic, myopathic, myelopathic and cerebropathic, respectively.

1. *Neuropathic*, are those atrophies dependent upon an inflammatory or degenerative condition of the peripheral nerves. If the atrophy follows a neuritis, as in acute simple neuritis, multiple neuritis, endemic neuritis, hemiatrophia-facialis, or a neuritis consequent to trauma, pressure, chemical irritation, or secondary to some inflammation of a neighboring organ, it is always accompanied by the general symptoms characteristic of nerve inflammation. (Trophic disturbances.) The atrophy is in most cases, of a severe type, local and shows marked electrical changes.

2. *Toxic Atrophies.*—Agents which have been instrumental in setting up a neuritic process and consequently wasting of the muscles are—alcohol, lead, arsenic, mercury, and bisulphide of carbon. The atrophy is generally limited to the extensor muscles, as seen in alcoholic paralysis, lead palsy, arsenical pseudo-tabes, and on eliminating the poison from the system, the atrophy sometimes disappears.

3. *After Infective Processes.*—Following upon an acute attack of diphtheria, variola, typhoid, typhus, cerebro-spinal meningitis, etc., atrophic changes may take place in some of the muscles of the body. The lesion is generally neuritic, the atrophy either the simple or hyaline degenerative, the latter especially in typhoid, variola and cerebrospinal meningitis. In typhoid fever, typical hyaline degeneration of the rectus abdominis, and adductors of the thigh may frequently be met with.

4. *Arthritic Atrophies.*—Following injury to joints, atrophy of the muscles moving that joint, but more especially the extensors, is often observed. If the hip joint is the seat of injury, there is atrophy of the glutei ; if the knee, the rectus femoris ; if the ankle, the gastrocnemius and soleus. The wasting is often quite pronounced and persistent, with little if any change in the electrical irritability and increased tendon reflexes. The seat of the lesion is purely hypothetical. Vulpian, Charcot and others believe that the articular centripetal nerves convey the irritation to the gray matter and particularly to the motor cells of the anterior horns, thence conveyed to the muscles of the joint through the efferent nerves.

The primary lesion in these neuropathic forms is to be sought for in the nerves supplying the affected muscles. The neuritis may be either interstitial, parenchymatous, or degenerative. In the interstitial form the medullary sheath is broken into fine granules of fat and débris and absorbed. The axis cylinder is swollen, degenerated, and may likewise be absorbed. The nuclei of the sheath of Schwann become swollen and proliferate, leading to the formation of new connective tissue, which, after the period of regeneration, constitutes the bulk of the nerve fiber. The perineurium and endoneurium also take part in this process and become converted into thick layers of connective tissue.

*Myopathic Form:* Sometimes called primitive or idiopathic, embraces a large class of atrophies which occur mainly in the young, are relatively rare, and as yet offer no clew as to the location of the primary lesion. Careful examination of the nerves and cord have in all cases proven negative, and it is the accepted opinion that the lesion is purely muscular—a myositis—and in some cases a fatty infiltration or lipomatosis.

Wide differences of opinion exist as to the proper classification of this type of atrophy. Although having a limited experience, nevertheless, the classification here appended seems to be the most plausible and capable of modification, should new facts and data be presented. Erb's juvenile form is taken as the type of this class. It is sometimes designated as the scapulo-humeral type, and begins in early youth and childhood. It is a progressive wasting and weakness of the muscles of the shoulder girdle, the upper arm and the back. The forearm, thigh and leg muscles remain intact for a long time. There may be present true or pseudo-hypertrophy of some muscles, as the deltoid, supra- and infraspinati. Fibrillar contractions and reaction of degeneration are never present. The reflexes are unimpaired.

Analogous to this type is the facio-scapulo-humeral type, first described by Duchenne as the *forme héréditaire*; but later more fully and minutely by Landouzy and Dejerine, in 1885. The wasting of some of the muscles of the face and hypertrophy of the lips give a peculiar tapir-mouth appearance to the patient, "facies myopathique." With this exception, this type of atrophy corresponds exactly with Erb's form, and is regarded by many as one and the same. Pathologically, the two show marked resemblances, there being atrophy and disappearance of some of the primitive bundles, with some hypertrophic fibers, and a slight increase of connective tissue and fat.

It has been questioned whether Leyden's hereditary form, first described in 1850, can stand as a clinical entity, or whether it must be classified under Erb's juvenile form. It appears in the majority of cases in males between the eighth and tenth year, and is decidedly hereditary in nature. It begins invariably with wasting and weakness of the muscles of the back and lower extremities. There are no sensory disturbances, no fibrillar contractions; electrical reaction is normal, patellar reflexes are present.

Another form is the pseudo-hypertrophic paralysis of Duchenne. Although hinted at years before by Bell, Meryon, Oppenheimer and Partridge, it remained for Duchenne, in 1861, to interpret correctly its clinical importance and establish it firmly in our nosology. It is no doubt hereditary, and occurs more frequently in boys than in girls. The important symptoms are weakness in the muscles of the leg and back, a waddling gait, an apparent increase in the size of the muscles of the calf, and sometimes thigh and calf. Furthermore, there is lumbar lordosis brought about by wasting of the muscles of the back and extensors of the thigh, some contractures, and a peculiar difficulty in rising from the ground. Repeated examinations of the nerves and cord have been unsuccessful, and hence the inference that the muscle itself is the seat of the lesion.

*Myelopathic Atrophies, or Atrophies dependent upon Lesions in the Spinal Cord.*—They may be acute or chronic. The acute forms are poliomyelitis infantilis (infantile paralysis) and poliomyelitis acuta adultorum. These two forms come more properly under the head of acute inflammatory processes of the cord, and belong only secondarily to our list of muscular atrophies. The chronic forms comprise most of the chronic affections of the cord. They are divided by Charcot, according to the seat of the lesion, into protopathic, where the insult resides solely and alone in the gray matter; and deuteropathic, where the gray matter is only secondarily affected. Under the first head we have the Duchenne-Aran, or hand type, characterized by wasting of the small muscles of the hand, the interossei, superficial and deep muscles of the thenar and hypothenar flexors and extensors of the forearm, biceps, deltoid, muscles of the back, shoulders and trunk. This type of atrophy is the original form of progressive muscular atrophy described by Duchenne and Aran in 1848 and 1850.

In 1886 there appeared simultaneously from Charcot and Marie in France, and Tooth in England, the description of another form of atrophy. Its mode of onset is by attacking the muscles of the lower extremities, the extensors of the toes, the small muscles of the foot, then the peronei, the calf muscles and later on the muscles of the thigh. The muscles of the hand and forearm may become involved after a lapse of years. In both of these forms fibrillar contractions, complete and partial reaction of degeneration, and absence of patellar and other reflexes are generally noted.

The pathology of these forms has been the subject of long and earnest controversy. The peripheral or myopathic origin was stubbornly held by Friedreich and the German school, while Cruveilhier, Charcot, Lockhart Clarke and others clung to the central or spinal origin theory. The latter is now the one universally accepted.

The anterior cornua of gray matter present the results of a subacute inflammatory process leading to complete or partial destruction of the ganglion cells, sclerotic changes in the neuroglia, blood-vessel changes, cell proliferation, etc. The contraction of the newly formed connective tissue may even lead to the formation of cavities in the gray matter. (See Plate, Fig. IV.) The anterior spinal roots are affected secondarily, likewise some of the efferent nerve fibers. Charcot's theory, then, is as follows: Atrophy of the muscular fibers is the direct result of irritation, which, beginning in the ganglion cells of the anterior horns, is propagated through the anterior spinal roots aud efferent nerves to the muscular fiber. Friedreich's theory was that the primary insult was a myositis with secondary changes as ascending neuritis of the peripheral nerve trunks, which terminated in a chronic myelitis.

The deuteropathic form comprises those affections in which the involvement of the gray matter of the cord is secondary. The atrophy following may be quite pronounced as in amyotrophic lateral sclerosis, syringomyelia, bulbar paralysis, glioma and other neoplasms of the cord. A careful examination is necessary at times to distinguish between the atrophy of these affections and progressive muscular atrophy. In locomotor ataxia, multiple sclerosis, diffuse myelitis and myelo-myelitis, the atrophy is less pronounced, inconstant and variable in its seat and intensity.

Lastly: *Cerebropathic Atrophies.*— Generally observed in the spastic paralyses of children and adults. The atrophy is limited to the paralized members, as in hemiplegia, paraplegia and diplegia. This class of atrophies comes more properly under the head of paralysis and calls for only passing notice.

### EXPLANATION OF PLATE.

FIG. I. Cross-section of a normal muscle. Zeiss' E objective, No. 1 eyepiece.

FIG. II. Simple degenerative atrophy of a muscular fiber. Zeiss E, No. 1 eyepiece.

FIG. III. Fatty infiltration and degeneration of a muscular fiber. Zeiss E, No. 1 eyepiece.

FIG. IV. Destruction of the antero-lateral group of ganglion cells, anterior coruna gray matter, spinal cord. The ganglion cells to the left (antero-median) are intact, while the antero-lateral have been replaced by cicatricial tissue. Zeiss E, No. 1.

The illustrations are reproductions of micro-photographs taken from specimens exhibited before the club.

## Figures and Tables

**Figure f1:**
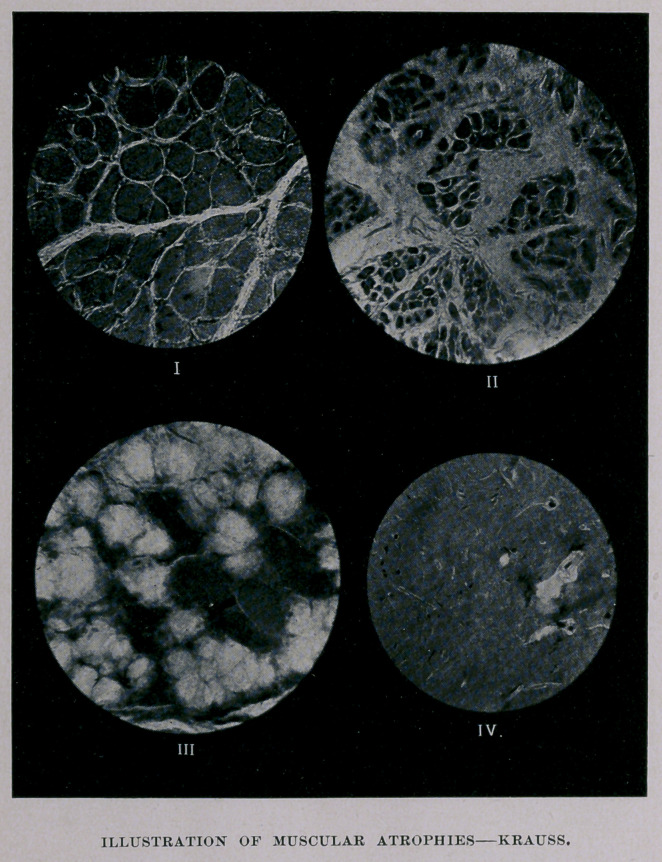


**Figure f2:**